# IVA cloning: A single-tube universal cloning system exploiting bacterial *In Vivo* Assembly

**DOI:** 10.1038/srep27459

**Published:** 2016-06-06

**Authors:** Javier García-Nafría, Jake F. Watson, Ingo H. Greger

**Affiliations:** 1 Neurobiology Division, MRC Laboratory of Molecular Biology, Cambridge, CB2 0QH, UK

## Abstract

*In vivo* homologous recombination holds the potential for optimal molecular cloning, however, current strategies require specialised bacterial strains or laborious protocols. Here, we exploit a recA-independent recombination pathway, present in widespread laboratory *E.coli* strains, to develop IVA (*In Vivo* Assembly) cloning. This system eliminates the need for enzymatic assembly and reduces all molecular cloning procedures to a single-tube, single-step PCR, performed in <2 hours from setup to transformation. Unlike other methods, IVA is a complete system, and offers significant advantages over alternative methods for all cloning procedures (insertions, deletions, site-directed mutagenesis and sub-cloning). Significantly, IVA allows unprecedented simplification of complex cloning procedures: five simultaneous modifications of any kind, multi-fragment assembly and library construction are performed in approximately half the time of current protocols, still in a single-step fashion. This system is efficient, seamless and sequence-independent, and requires no special kits, enzymes or proprietary bacteria, which will allow its immediate adoption by the academic and industrial molecular biology community.

Molecular cloning is at the heart of biomedical and biotechnological research, fundamental to protein structure-function studies, protein engineering and synthetic biology^1^,^2^,^3^. Since the advent of the polymerase-chain reaction (PCR)^4^, cloning has involved selective PCR amplification and modification of DNA segments, which require directed assembly into a plasmid carrier for propagation in *E. coli*. Traditionally, restriction enzymes and ligases have been used to direct the assembly of DNA fragments^1^; however, their sequence-dependency and laborious protocols led to the development of new alternatives, such as: PCR-only^5^,^6^,^7^,^8^, ligation-independent cloning (LIC)^9^, recombination-based^10^,^11^ and multi-enzyme construction methods (such as Gibson^12^, In-Fusion^13^ and USER^14^). LIC, multi-enzyme construction and some recombination-based approaches (such as SLiCE^10^) rely on *in vitro* enzymatic treatment of DNA fragments for assembly. While PCR-only methods eliminate the need for such enzymatic treatment, they involve multiple rounds of PCR and DNA purification. Both cases involve lengthy, hands-on protocols. *In vivo* assembly, where the bacterial host performs the fusion of DNA fragments, would eliminate the need for multiple steps and reagents, providing significant advantages over all current methods. The advantages of *in vivo* recombination have led molecular biologists to use the yeast gap-repair cloning system^15^, despite the hurdles associated with eukaryotic work. *In vivo* assembly in *E. coli* has previously been limited to the use of strains with enhanced recombinase activities, such as recA^16^ and phage recombinases Red/ET^17^,^18^, yet these bacterial strains enhance plasmid instability or require specialised preparation of competent cells. As a consequence, strains with reduced recombinase activities (e.g. recA knockouts) are universally used for molecular cloning.

The presence of a recA-independent homologous recombination pathway in *E. coli* was reported more than 20 years ago^19^,^20^,^21^, but has been neglected until recently, except for sporadic use in specific high throughput applications^22^,^23^. The pathway is mostly uncharacterised but is most efficient at recombining linear DNA fragments, likely acting through an annealing mechanism^20^,^24^, although alternative mechanisms have been suggested^25^,^26^. Conveniently, the recA-independent pathway is responsible for the recombination of short overlapping sequences^19^, whereas the recA system requires longer homologous DNA stretches (>150–300 bp). The pathway’s short homology requirements, ubiquitous presence in laboratory *E. coli* strains (in our and others’ experience^27^,^28^,^29^) and reduced compromises on plasmid stability, make it an optimal tool for molecular cloning. Recently, this potential has begun to be realised, both *ex vivo*^27^ and *in vivo*^28^,^29^,^30^, where gene cloning has been performed using *in vivo* assembly in *E. coli*. However, protocols are still laborious and require commercial kits, presenting a limited advantage over widely used cloning methods using *in vitro* enzymatic assembly, such as In-Fusion and Gibson assembly.

In this study we exploit recA-independent homologous recombination to develop a complete cloning system: *In Vivo* Assembly (IVA) cloning. All cloning procedures (insertions, deletions, mutagenesis and sub-cloning) can be performed and combined at will, using a single universal protocol consisting exclusively of a single-tube PCR followed by *Dpn*I digestion and transformation.

## Results

To implement IVA, we first optimised primer design and investigated the efficiency of the recA-independent recombination pathway to assemble multiple DNA fragments. This allowed us to develop a complete cloning system capable of performing both single and multiple modifications with unprecedented speed and simplicity, and facilitating the construction of any desired plasmid from a single PCR. We then investigated the potential of this system for plasmid library construction, using the assembly of multiple DNA fragments to create a small mammalian expression library. We provide examples from our work using cDNAs of ionotropic glutamate receptors (AMPA receptor subunits GluA1–4)^31^ and their associated membrane proteins (TARP γ2^32^ and GSG1L^33^), in a variety of mammalian expression vectors.

### Method overview and primer design

**Figure 1:**
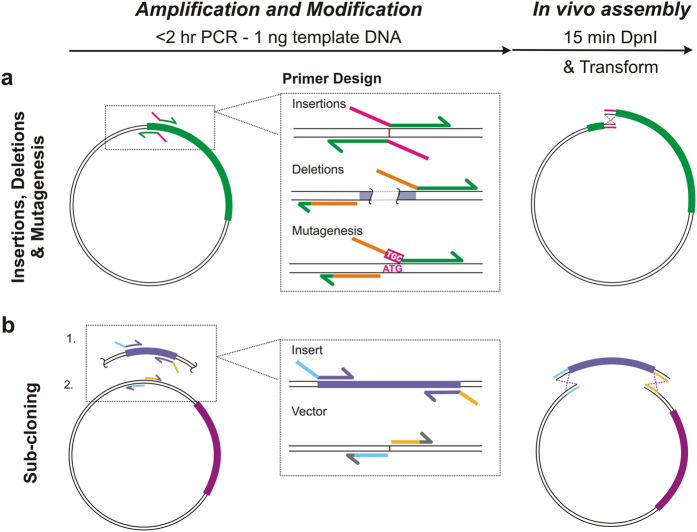
Method overview and primer design optimisation. **(a)** Schematic of the universal IVA cloning protocol consisting of a single PCR reaction, producing homologous linear ends, followed by *Dpn*I digestion and transformation, where amplified DNA is assembled *in vivo* by recombination. Primer design is shown for each type of basic modification: insertion, deletions, site-directed mutagenesis and sub-cloning. For insertions, the new sequence is best included in Fw and Rv primers, acting as the homologous region (magenta). For deletions, the overlap can be incorporated in any one primer, homologous to the other primer (orange) with primers straddling the undesired region (grey). Mutagenesis is similarly performed, inversely amplifying outside the undesired codon (ATG), with the replacement encoded in the forward primer (TGC). **(b)** Sub-cloning involves the amplification of both vector and insert in a single tube with homologous regions to directionally control assembly (blue and yellow).

IVA cloning uses *in vivo* assembly of PCR amplified DNA fragments, guided by short homologous flanking regions that are fused together by recombination. All cloning procedures for single or multi-site modifications, proceed through a single-step PCR, template DNA digestion with *Dpn*I (an endonuclease specific for methylated DNA) and transformation (Fig. 1). As outlined in Fig. 1, all DNA modifications and homologous regions are introduced at the 5′ end of the primers. Insertions (of short sequences that can be included within the primers), deletions and site-directed mutagenesis proceed through inverted PCR with primers binding astride the modification site (Fig. 1a). For insertions, it is cost-optimal to include the extra sequence in the overlapping regions of both Forward (Fw, 5′-3′) and Reverse (Rv, 3′-5′) primers, acting as the homologous region (Fig. 1a). Deletions require inverted primers flanking the undesired region, amplifying outwardly, with the Fw primer containing a region homologous to the Rv primer. Similarly, mutation primers flank the undesired codon, with the new sequence encoded in the Fw primer. Sub-cloning uses PCR of the vector at the location for insertion, while the insert is amplified independently in the same tube, with homologous regions at both linear ends (Fig. 1b). These regions may be included in either the vector or the insert primers. Due to the inverted nature of the primer design, multiple modifications can be performed, simply by combining primers for single modifications in the same PCR tube (described in detail below). Hence, any combination of plasmid modifications may be performed using the same protocol and primers as for single-site protocols.

### Method and primer design optimisation

**Figure 2:**
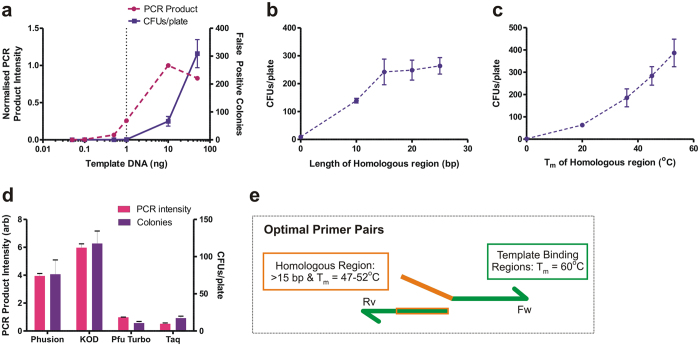
Method and primer design optimisation. **(a)** Performing PCR with no homologous regions highlights potential false positives arising from template DNA. Increasing template DNA increases the number of colonies produced on transformation (■ purple), independent of PCR amplification (● magenta). 1 ng regularly produces 0 colonies, yet gives substantial PCR amplification (dashed line). **(b)** Relationship between increasing length of homologous regions (constant T_m_) and colony yield shows little length dependence of recombination above 15 bp. **(c)** Increasing the T_m_ of homologous regions increases the colony yield and hence recombination efficiency. **(d)** Bar chart indicates that IVA cloning colony yield (purple) is reliant on amount of PCR product (magenta) independent of the type of PCR polymerase. **(e)** Properties of optimum primer design to maximise recombination efficiency. Homologous regions are included in 5′-end of primers, homologous to a region (orange) of the partner primer. Template binding regions are shown in green.

False positive colonies can originate from undigested template DNA. To maximise the percentage of clones containing the desired product, we first identified the highest amount of PCR template plasmid that resulted in no false positive colonies after *Dpn*I digestion. To do so, we performed a deletion of the GluA3 AMPAR N-terminal domain (NTD) coding region in the pRK5 plasmid with increasing amounts of template plasmid (0–50 ng) (Supplementary Table S1, OPT1 primers). Using primers devoid of overlapping regions, and therefore unable to recombine (no true positives), all colonies originated from undigested template DNA. As expected, the number of colonies correlated with amount of template used, and not with amplification intensity (see 50 ng point), suggesting that colonies originated exclusively from undigested template plasmid (confirmed by restriction analysis and Sanger sequencing) (Fig. 2a and Supplementary Fig. S1). We found that 1 ng of template DNA provided the best PCR product amplification when false positives rarely occur.

To maximise the level of recombination, we studied the effect of both length and binding strength (melting temperature, T_m_) of the homologous regions. For both assessments, primers were designed to remove the GluA3 NTD coding region in the pRK5 plasmid. The number of colony-forming units after amplification (using overlaps from 10 to 25 bp in length at a constant T_m_ of 40 °C) plateaued at lengths greater than 15 bp (Fig. 2b) (OPT2–5 primers). Altering binding strength (at constant 15 bp length) showed increasing recombination (greater CFUs/plate) with higher T_m_ (Fig. 2c) (OPT6–9 primers). In both cases, no difference in level of amplification was found between the primers, with results exclusively dependent on the efficiency of recombination (Supplementary Fig. S1).

To determine whether there is any preferential enzyme requirement for this system, we tested four commercial DNA polymerases (Phusion, KOD Hot Start, Pfu Turbo and Taq polymerase). The same GluA3-NTD deletion was performed with each enzyme (OPT8 primers). PCR efficiency was variable between enzymes (Fig. 2d and Supplementary Fig. S1) and colony number correlated with the level of amplification (Fig. 2d), meaning that levels of DNA assembly are influenced by PCR amplification efficiency and not the specifics of enzyme activity.

As a result of the optimisation, 1 ng of template DNA was used throughout to minimise false positives. In order to avoid primer-dimer formation through overlaps, optimal homologous regions should be designed to be at least 15 bp in length with a T_m_ of ~47–52 °C (normally resulting in lengths of 15–20 bp) (Fig. 2e). Phusion polymerase was used at all times due to its high PCR efficiency and fidelity. Although KOD polymerase yielded higher amplification, it has lower fidelity^34^, an essential property when amplifying whole plasmids with minimal errors.

### Simplifying basic molecular cloning procedures using IVA

#### Insertions and deletions

**Figure 3:**
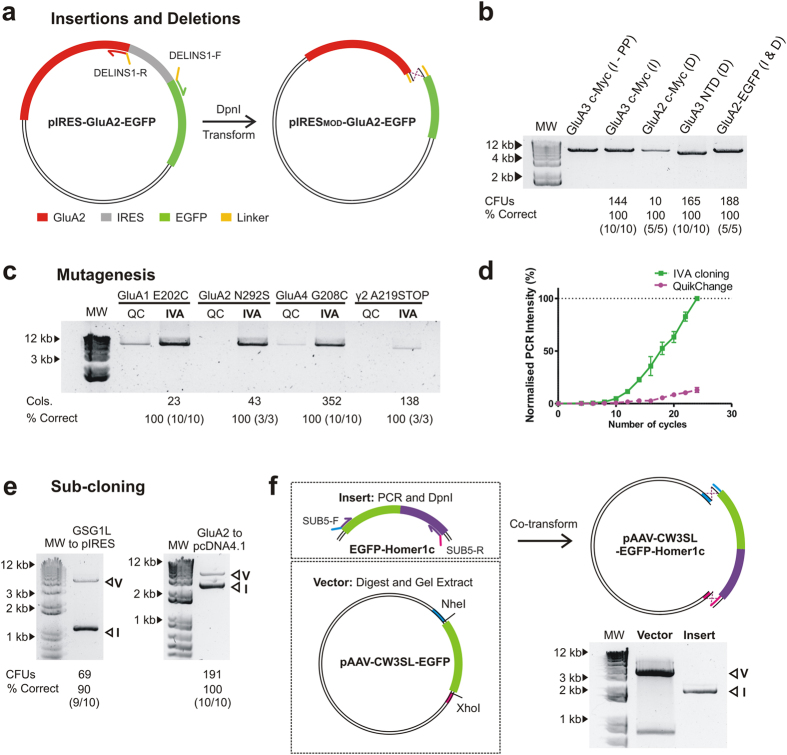
Basic molecular cloning procedures using IVA cloning. **(a)** Schematic depicting the simultaneous deletion of an IRES cassette (grey) and insertion of a linker sequence (yellow) in the GluA2-pIRES-EGFP vector. **(b)** Agarose gel showing the resulting amplification of insertions (I) and deletions (D). These include (Lane 2) insertion of a myc-tag at the N-terminus of GluA3 using phosphorylated primers (PP), and (Lane 3) IVA primers, (Lane 4) deletion of an N-terminal myc-tag in GluA2, (Lane 5) deletion of the N-terminal domain of GluA3 and (Lane 6) construction of a fusion GluA2-EGFP tandem construct by deleting the IRES cassette and introducing a linker. Number of colonies produced on transformation, and the percentage of colonies tested that contain the correct plasmid is shown below. MW  =  1 kb DNA ladder. **(c)** Agarose gel of PCR products providing a comparison between IVA and QuikChange TM mutagenesis primers. An enhancement of the intensity is seen for IVA primers in all cases. Number of colonies and percentage of correct clones for IVA cloning are shown below. **(d)** Cycle-by-cycle comparison of the PCR product formation between IVA (■ green) and QuikChange^TM^ (● magenta) for the GluA4 G208C mutation over 24 cycles of PCR (normalised to maximum value as 100%, n  =  3). The increased PCR yield of IVA is appreciable. **(e)** Agarose gel electrophoresis visualisation of PCR products for sub-cloning examples (GSG1L coding region into pIRES-mCherry and GluA2 coding region into pCDN4.1/TO) each showing two independent amplifications (Vector: V, Insert: I). Colony yields and percentage correct are shown below. **(f)** Alternative strategy for vectors not amenable to amplification, shown with the cloning of EGFP-Homer1c (Insert), subject to PCR, *Dpn*I treatment and PCR purification, into the adeno-associated virus vector pAAV-CW3SL-EGFP (cut with *Nhe*I and *Xho*I, and gel purified. Agarose gel visualisation of vector post-digestion identifies gel purified fragment (V) alongside PCR amplified Insert (I).

Insertions and deletions are common modifications, used for adding or removing protein tags, domains or gene promoters. The current most popular method is inverted PCR using 5′-phosphorylated primers, which requires expensive primers, PCR purification and enzymatic ligation of amplification products. IVA cloning eliminates such steps, kits or phosphorylated primers. As examples, we performed: 1) insertion of an N-terminal c-Myc tag in the pRK5-GluA3 plasmid (30 bp) (INS1 primers), 2) deletion of a N-terminal c-Myc tag in a pIRES2-EGFP-GluA2 plasmid (30 bp) (DEL1 primers), 3) deletion of the NTD of GluA3 in the pRK5 vector (1140 bp) (DEL2 primers) and 4) construction of a GluA2-EGFP fusion construct by replacing the IRES cassette from the pIRES-EGFP-GluA2 plasmid with a linker sequence (DELINS1 primers) (Fig. 3a). No effect on amplification was seen when comparing IVA overlapping primer design to phosphorylated primers (INS2 primers) (insertion of a c-Myc tag, Fig. 3b), yet protocols are markedly shortened. Significant numbers of colonies (10–188) were formed when transfected in bacteria (produced in-house), with a transformation efficiency of 3 × 10^6^ CFU/μg of pUC18, with 100% of colonies containing the correctly modified product in every case (Sanger sequencing of 5–10 colonies per plate) (Fig. 3b).

#### Site-directed Mutagenesis

Site-directed mutagenesis is an essential technique for protein engineering and protein structure-function studies. A variety of methods have been designed (reviewed in^35^) but the commercial QuikChange™ Mutagenesis is the most widely implemented. QuikChange™ relies on amplifying the whole plasmid with fully overlapping primers containing the desired mutation. The nature of primer design favours primer-dimer formation, limiting the amount of amplified product and giving rise to false positives (only 80% of clones correct). Several studies have shown that improved amplification was achieved by slightly displacing the binding region of the primers^36^,^37^. Primer design for site-directed mutagenesis using IVA cloning allows full displacement of the primers with the modified base pairs outside the template binding region (Fig. 1a and Supplementary Fig. S2). This eliminates primer-dimer formation and mispriming due to incorrect base pairings.

Here we show examples of four mutations in protein coding genes: GluA1 E202C, GluA2 N292S, GluA4 G208C and TARP γ2 A219Stop (MUT1–4 primers respectively, using the vectors pIRES2-mCherry-GluA1, pIRES2-EGFP-GluA2, pRK5-GluA4 and pGW1-TARP γ2). Their amplification yield is compared to the QuikChange™ mutagenesis method (MUT5–8 respectively). Amplification efficiency was consistently higher using IVA primers (Fig. 3c), which obtained an exponential (Fig. 3d) and cleaner amplification (Supplementary Fig. S2 - low molecular weight smearing with QuikChange™). Significant colony numbers (23–352) and 100% correct mutagenesis (of 5–10 colonies tested) were obtained for all IVA examples. For TARP γ2 A219Stop, both primer designs gave lower amplification than in other examples, yet IVA primers still showed enhanced amplification. Since amplification levels generally correlated with colony number, QuikChange^TM^ examples were not transformed due to their poor amplification.

#### Sub-cloning

The current most favourable routes for sub-cloning are homology-based (e.g. In-Fusion^13^ or Gibson Assembly^12^), and require separate PCRs for insert and vector, DNA purification and enzymatic assembly. Sub-cloning with IVA is achieved by amplifying both insert and vector from two plasmids (insert containing and target vector) in a single PCR. We show examples of: 1) sub-cloning of GSG1L, into the pIRES2-mCherry vector and 2) sub-cloning of the GluA2 coding region from the pIRES2 into the pcDNA4/TO vector. Overlapping regions were added to the insert in both cases (SUB1–2 primers respectively in Supplementary Table S1). Two amplification products, corresponding to vector and insert are produced by PCRs (Fig. 3e) which were directionally assembled *in vivo* with 90% and 100% respectively containing the desired insert (10 colonies tested).

Occasionally, high secondary structure formation, excessive length (i.e. Bacterial Artificial Chromosomes [BACs] and Yeast Artificial Chromosomes [YACs]) or vectors containing repeats (i.e. adeno-associated virus [AAV] vectors) may prevent vector amplification. While this can generally be overcome by using DMSO or betaine in PCRs^3^,^38^, certain vectors are not amenable for amplification. In such cases sub-cloning can be performed by restriction digestion of the vector at chosen locations and co-transformation with the desired insert, which contains regions homologous to termini of the digested vector (Fig. 3f). As an example, we have sub-cloned EGFP-Homer1c into the pAAV-CW3SL-EGFP plasmid, digested with *Nhe*I and *Xho*I. Homologous recombination is able to assemble the linear fragments, producing 55 colonies with 60% containing the desired plasmid (12/20 colonies). Transformation of the digested vector alone produced 25 colonies, with recombination at inverted terminal repeat (ITR) sequences of the AAV vector likely causing this high number of incorrect colonies.

### Multi-site genetic modifications

**Figure 4:**
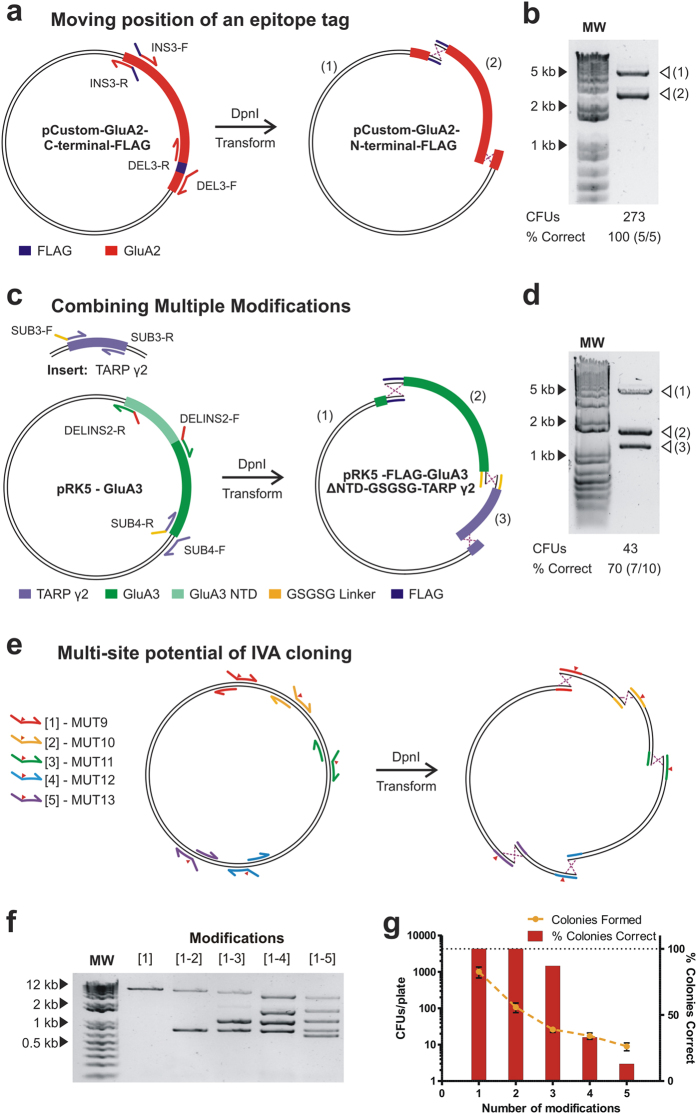
One-tube multi-site modifications using IVA cloning. **(a)** Schematic for multi-site modification whereby the position of a FLAG-tag (purple) is exchanged from the C- to the N-terminus of GluA2 (red) coding region in a CMV-based custom plasmid. The combination of deletion and insertion primers produces two amplification products after PCR. **(b)** The corresponding fragments (1 and 2) are visualised by agarose gel electrophoresis. **(c)** Schematic detailing multiple plasmid modification of GluA3-pRK5 vector in one tube. One set of primers a) deleted the N-terminal domain of GluA3 and b) inserted a FLAG-tag, while a second set of primers a) sub-cloned the TARP γ2 coding region (from a second vector) at the end of GluA3 and b) inserted a GSGSG linker to create a fusion construct. Together, these primers amplify three independent fragments, which are shown on an agarose gel **(d)**. **(d)** Testing the number of multiple modifications that IVA cloning can perform simultaneously. Increasing number of *Xho*I restriction sites were created in the pRK5 plasmid using mutagenesis primers. Site of mutation is indicated by ▼. **(f)** PCR produced increasing numbers of bands corresponding to the number of modifications (1 to 1–5). **(g)** The number of colonies produced (yellow) and the percentage of correct clones (red) decreased with more modifications (n  =  3–5).

Performing multiple modifications on a plasmid is regularly desired, with applications in antibody maturation, protein thermostabilisation and gene repair after synthesis. Current strategies are laborious and time consuming, involving multiple rounds of individual modifications. Mainly, protocols for multi-mutagenesis have been reported^39^,^40^,^41^,^42^,^43^ which involve multiple steps and/or expensive primers. Although Gibson cloning has been proposed to enable multiple modifications of any kind^41^, investigators normally turn to commercial gene synthesis for complex procedures. IVA cloning permits multi-site modifications (insertion, deletion, mutagenesis and sub-cloning) merely by combining primers for single modifications in the same PCR reaction. Due to the inverted nature of the primer design, whereby primers amplify outwardly from each other around the vector, independent amplification products will be formed between each primer-binding site: one Fw primer will combine to amplify a segment with the Rv from the downstream modification. Upon transformation, the unique overlapping sequences will guide assembly of all fragments to form the desired propagative plasmid. As more primers are added, the circular plasmid amplifies in increasing smaller segments, reducing the PCR extension times required. This approach allows simultaneous modifications of any type in a rapid and simple manner. To demonstrate this principle we first swapped the position of a FLAG-tag from the C-terminus of a GluA2 coding sequence to the N-terminus in a CMV-based custom vector (Fig. 4a). Primers were designed as per single modifications to remove the C-terminus tag and insert it at the N-terminus (INS3 and DEL3 primers in Supplementary Table S1). Upon PCR, two amplification products were seen (Fig. 4b) producing 273 colonies on transformation with 100% containing both modifications (5/5 colonies tested).

More complex modifications can be generated in a similar fashion. To provide an example, we built a pRK5-FLAG-GluA3-ΔNTD-GSGSG_linker_-TARP γ2 tandem construct, encoding two modified proteins fused by a peptide linker, starting from the pRK5-GluA3 and the pGW1-TARP γ2 plasmids. For this we designed primers 1) to remove the N-terminal domain coding region of GluA3 and replace it with a FLAG-tag (DELINS2 primers), and 2) to sub-clone the TARP γ2 coding region after the GluA3 coding region, with a GSGSG linker at the fusion site (SUB3 and 4 primers, linker encoded in primers as per insertions) (Fig. 4c). A ~1.5 hr PCR was required, producing three DNA fragments (Fig. 4d). 43 CFUs/ plate were produced, of which 70% (7/10 colonies) contained the correct sequence.

#### Assessing the multi-site potential of IVA cloning

In order to test the number of simultaneous modifications that can be performed, we designed primers to introduce up to five *Xho*I restrictions sites in the pRK5-GluA3 plasmid (MUT9–13 primers) (Fig. 4e). PCR reactions were set up with increasing numbers of mutational primers (up to five), as seen by the corresponding number of bands produced after amplification (Fig. 4f). Decreasing colony yields and percentage of correct clones were observed as the number of mutations (and therefore recombination events required) increased (Fig. 4g). This was seen reliably with narrow standard deviations among replicates. 100% of colonies were correctly modified for single and double mutations, consistent with previous examples of single modifications (one recombination event) and sub-cloning (two recombination events) examples described above. For three simultaneous modifications, 87% of colonies contained all mutations, in line with tandem construct production. Four and five mutations could be successfully produced with 33% and 13% of colonies being correct.

### Multi-fragment assembly and library construction

**Figure 5:**
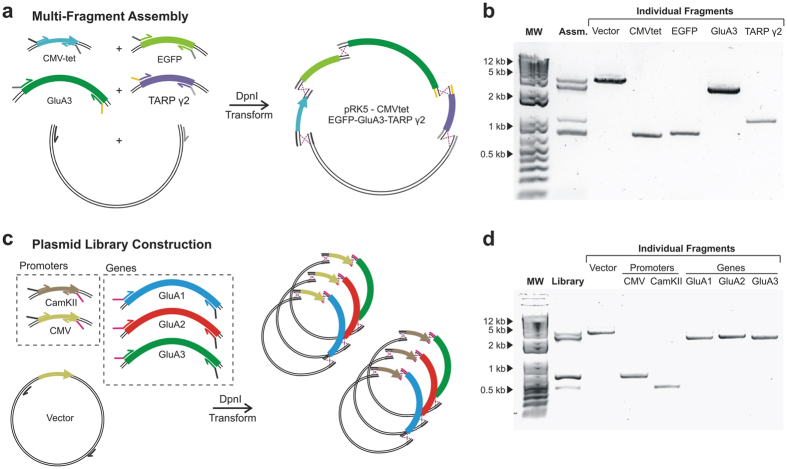
Single-tube multi-fragment assembly and library construction using IVA protocols. (**a)** Schematic of a multi-fragment assembly where five independent fragments (CamKII, EGFP, GluA3 and TARP γ2 coding regions together with the pRK5 vector) were amplified in one PCR and assembled *in vivo*. **(b)** The amplification result is shown by agarose electrophoresis (Lane 2). Individual fragments were independently amplified to facilitate identification (Lanes 3–7). **(c)** Schematic of mammalian expression library construction. Two promotors (CamKII and CMV) and three genes (GluA1, GluA2 and GluA3 coding regions) where amplified in a single tube alongside the pRK5 vector. Assembly is guided by specific homologous regions that are shared within promotors and within genes. **(d)** Agarose electrophoresis resulting from the amplification in a single tube (Lane 2) with individual fragments shown (Lanes 3–8) to aid in the identification.

Since the simultaneous introduction of five mutations required the *in vivo* assembly of five separate DNA fragments, we reasoned that the same approach could be applied to the assembly of multiple fragments originating from different plasmids. For this we aimed for a fusion construct containing a tetracycline-inducible CMV promoter (CMVtet) (625 bp), EGFP (720 bp), the GluA3 coding region (2667 bp) and the TARP γ2 coding region (972 bp), all cloned into a pRK5 backbone (3573 bp) (Fig. 5a). These DNA fragments were independently PCR amplified in the same tube from different template plasmids (pRK5-GluA3, pcDNA4/TO, pN1-EGFP and pGW1-TARP γ2) in a 1.5 hr reaction (Fig. 5b) (ASS1–5 primers). Unique homologous regions were designed to drive the ordered assembly of a pRK5-CMVtet-EGFP-GluA3-TARP γ2 construct. After PCR, *Dpn*I digestion and transformation, the construct was successfully produced in 14% of the clones (2/14 colonies tested), in line with the efficiency seen for five simultaneous mutations.

DNA libraries with randomised sequences are important tools for protein evolution or selection of nucleotide aptamers. The ability to combine multiple different fragments with high efficiency permits construction of plasmid libraries, where many alternative DNA fragments are incorporated. Mammalian protein expression often requires optimisation, as expression levels are low and sample dependent. The case-by-case optimisation of constructs with different vector properties seems to be among the most popular strategies to overcome the problem^44^,^45^,^46^,^47^. The simplicity of IVA cloning ideally lends itself to such applications. As a proof of principle, we built a small mammalian expression library where promoters and genes were randomly shuffled and sub-cloned in a vector backbone. Two promoters (CMV and CamKII), three genes (GluA1, GluA2 and GluA3 coding regions) and the pRK5 backbone were amplified in a single PCR (vector and CMV from pRK5-GluA4, CamKII from pCamKII-EGFP-Homer and GluA1–3 from pIRES-GluA1, pIRES-GluA2 and pIRES-GluA3). Common overlapping regions were introduced between backbone and promoters, promoters and genes and genes and backbone, such that plasmids will be formed randomly of any promoter and any gene (LIB1–6 primers) (Fig. 5c). The standard IVA protocol was applied, using a 1.5 hr PCR amplification of all fragments in one tube (Fig. 5d). In order to assess the efficiency of library formation we calculated the expected number of sampled colonies required to find all constructs. Assuming 87% of colonies will contain a properly assembled plasmid (three recombination events), 20 colonies would need to be examined in order to find all possibilities with 95% confidence (0.95  =  1 − (1 − f)^n^), where “f” is the frequency of the least occurring construct and “n” the number of colonies required. Sampling 15 colonies successfully provided the full plasmid library, highlighting the efficiency and versatility of IVA.

## Discussion

**Figure 6:**
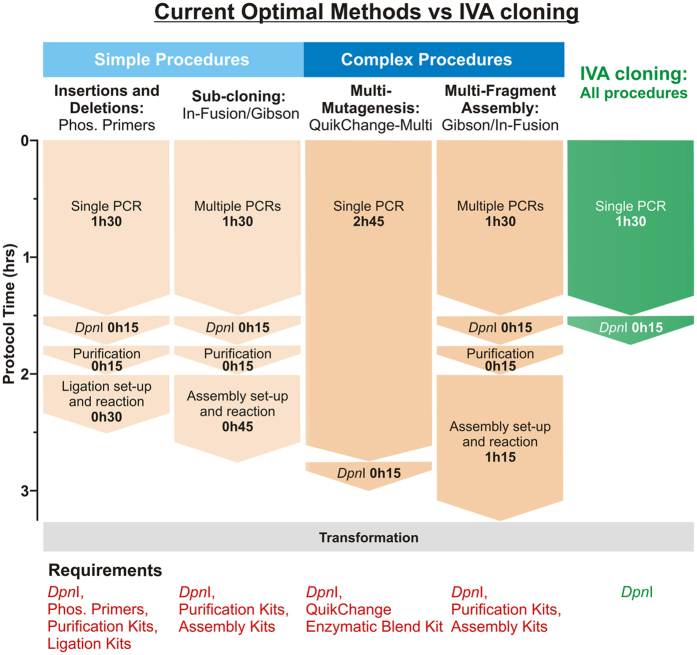
Comparing IVA with current optimal protocols for each cloning procedure. Optimal methods for each type of cloning procedure have been selected (orange) for comparison with IVA (green). Labour time and requirements are shown for each example, with the universal IVA protocol significantly outperforming all methods, becoming the best option for all procedures. Of special importance are multisite applications (‘Complex Procedures’), where IVA halves the time required by the next best method and eliminates costs associated with enzymatic assembly and DNA. Furthermore, the IVA multi-site protocol surpasses optimal methods for performing single modifications (‘Simple Procedures’). All protocols require transformation into *E. coli* (grey). Contrasting with other methods, IVA only requires *Dpn*I (‘Requirements’). (Phos.  =  phosphorylated).

The IVA system provides key advantages over current molecular cloning methods. Firstly, the single-tube, single-step PCR protocol, together with the elimination of enzymatic treatment or purification kits, turns this system into the fastest and cheapest cloning method currently available. In Fig. 6, we compare current optimum cloning procedures to the IVA protocol. Considering hands-on time, protocol length and reagents required, IVA cloning readily outperforms the current most favourable method for each cloning procedure in these aspects. Particularly significant is the improvement in complex procedures, where protocol lengths are almost halved (IVA: 1h45, Gibson Assembly: 3h15) and hands-on time is reduced by ~70% (Fig. 6). IVA cloning allows complex procedures (i.e. multi-fragment assembly) to be performed faster than other favourable methods can achieve simple procedures. While being rapid and cost-effective, IVA cloning is also efficient, sequence-independent, directional and seamless.

IVA is a complete, universal cloning system that can perform all cloning procedures using a single protocol. While recent homology-based methods, such as Gibson Assembly and In-Fusion are suitable for assembling multiple DNA fragments, their protocols for single modifications are less favourable to alternative methods (e.g. phosphorylated primers for small insertions). Therefore, different systems and primer design are applied to individual procedures, which are then not compatible in future cloning strategies (e.g. combining phosphorylated primers for insertion and QuikChange^TM^ for mutagenesis is unfeasible). IVA primers designed for any single modification are readily usable for combinatorial applications making it the most versatile system to date.

The Golden Gate cloning and MoClo systems are particularly useful for library formation and gene shuffling^48^,^49^,^50^. These rely on type IIs restriction endonucleases and have been reported to allow the one-pot construction of shuffled libraries of up to ~20,000 variants, yet the method relies on availability of restriction sites or initial cloning into appropriate vectors. IVA cloning has the potential to perform such applications without the need of specific restrictions sites. However, since the IVA method requires PCR, there are some limitations on the size of DNA fragments that can be amplified.

IVA cloning exploits a recA-independent recombination pathway, which is emerging as a powerful tool in DNA manipulation. Initial reports of this bacterial pathway and its application to cloning were not rapidly adopted, possibly due to the simultaneous reporting of *in vivo* cloning using bacterial strains expressing phage recombinases^51^, which are now widely used for genome engineering^52^. While the recA-independent pathway has recently been utilised as a cloning tool in AQUA cloning, the protocols involved for its use *in vivo* require multiple PCRs, gel extraction, mixing of DNA fragments and incubation prior to transformation^28^,^29^,^30^. Although cost-effective when compared to enzymatic assembly methods, protocols are significantly longer (3h30 from set-up to transformation). IVA cloning is efficient without such requirements, providing a fast, versatile and cost-effective system that outperforms all current cloning methods. Performing separate PCR reactions may increase efficiency when assembling >5 DNA fragments, since longer homologous regions can be included that could cause primer annealing problems if mixed in a single tube. Conversely, for library creation, individual PCR amplifications of gene variants would be completely unfeasible when using multiple orders of magnitude, whereas the single-tube IVA method would offer no greater difficulties. While IVA is advantageous for almost all cloning applications, multiple modification of very large plasmids, such as BACs, which cannot be PCR amplified, would require different a different approach.

The recA-independent recombination pathway is present in laboratory *E. coli* strains regularly used for cloning. Since specialised strains do not need to be obtained, IVA cloning can immediately be adopted. Although we have used XL-10 Gold cells throughout this study, we have successfully tested DH5α, Mach1 and Stbl3 strains with similar success, and recent reports have shown efficient recombination using extracts from a variety of standard bacterial strains extracts (TOP10, NEB5α, NEB10β, BL21 (DE3) and JM109)^27^,^28^, highlighting the ubiquity of the pathway. We believe that the number of modifications attainable is dependent on the transformation efficiency of the bacterial strains, as seen by lower colony yields when using our homemade cells. Highly competent strains or better transformation methods could enhance IVA cloning to allow even more complex assemblies.

Although the recombination mechanism is still unknown, it is clear that *in vivo* DNA assembly using common bacterial strains is a powerful tool for molecular cloning. In our experience, when PCR amplification is successful, correctly assembled product is invariably formed. Therefore, optimisation of PCR conditions (i.e. varying primer annealing temperature or using additives such as betaine or DMSO) are the key routes for troubleshooting IVA cloning. “Incorrect” recombination events were never observed with single or double modifications, but as the number of DNA fragments to assemble is increased, non-specific recombination events can produce colonies, contributing to the lower percentage of clones that contained the desired product (see Fig. 4g). However, the percentage of correct clones observed was consistent for different examples that require equal numbers of recombination events. For example, a reliable percentage of correct clones were produced for the sub-cloning examples, the tag-swap and double mutation (90–100%). We observe that performing multi-site modifications with target sequences separated by less than 150 bp can be problematic, however, inexpensive primers of up to 110 bp are now available, which could include both modifications and would allow such procedures to be conducted using a single recombination event.

Recombination is not the only mechanism that has so far been proposed for this cloning strategy^25^,^26^,^30^. One alternative suggestion is that 3′–5′exonuclease activity of the PCR polymerase creates single-stranded 5′- overhangs at fragment ends, which allow the formation of nicked circular plasmids that can be repaired after transformation. We provide strong evidence against this hypothesis. Firstly, Taq polymerase, lacking 3′–5′ exonuclease activity, can be successfully applied to IVA. Secondly, in our hands, promoting insert and vector annealing prior to transformation did not enhance colony formation. Finally, successful clones are formed if vector DNA is linearised using restriction enzymes, without single-stranded overhangs that can anneal, and co-transformed with the insert. As insert and vector are mixed on ice at transformation, plasmid assembly can only occur *in vivo*, likely as a result of recombination, an observation supported by other studies^28^,^30^. Insights into the recA-independent pathway will clarify the DNA assembly mechanism and can provide routes for further improvement of the IVA system.

Interestingly, a recent mechanistic study of the QuikChange^TM^ mutagenesis method^37^ shows that, in contrast to previous evidence, this popular method actually proceeds through exponential (rather than linear) amplification. The authors hypothesise that overlaps are formed at linear ends that must be fused together by an uncharacterised recombination mechanism. We support this hypothesis, as we can fit an exponential curve (R^2^  =  0.86) to the amplification of the GluA4 G208C mutant using QuikChange^TM^ primers (Fig. 3d and Supplementary Fig. S2). Overall, the QuikChange^TM^ site-directed mutagenesis method appears to proceed through the recA-independent recombination pathway, however primers have not been optimised for that purpose. Our primer design lies at the optimal theoretical point, with the mutation lying fully outside the primer annealing region, giving greatest control over the primer-template T_m_ and fully avoiding primer-dimer formation, while optimally utilising the recombination pathway.

Using IVA primer design and the one-tube strategy, highly complex cloning protocols can be performed rapidly and effortlessly, with minimal hands-on time. We envisage that applications of the system would have an immediate impact on different fields, from fundamental biochemical research to protein engineering and synthetic biology. We have demonstrated how IVA cloning provides a platform for simplified randomisation, while the minimal hands-on time is of critical importance for high-throughput studies. This will greatly benefit protein engineering projects, for example allowing the simultaneous randomisation of a loop length and a saturation site-directed mutagenesis. Synthetic biology is now facilitated by developments such as BioBricks^53^, where DNA blocks can be purchased to be assembled at the user’s preference. Advances in directional assembly of DNA fragments are key to progress. Several popular cloning methods are currently applied to such an assembly, for example Gibson cloning^12^, USER^14^ and In-Fusion^13^; however, IVA cloning has no enzymatic assembly requirements and reduces labour time. Molecular cloning is a core technique in biomedical research and IVA cloning will simplify construct preparation for all molecular biologists. Fundamental research is gearing towards the study of more technically challenging protein systems, such as protein complexes, membrane proteins and unstable proteins. Bottlenecks are regularly found during protein expression, purification and stability, and construct design has become a key tool to overcome these barriers. IVA cloning provides a platform where multiple plasmid modifications can be performed and combined as desired, eliminating a significant barrier to ideal experimental design and unifying molecular cloning to a single protocol.

## Materials and Methods

### *E. coli* strains and reagents

*E. coli* XL10-Gold competent cells were used throughout, either prepared by the Rubidium Chloride method or commercial (Agilent) (efficiency of ~3 × 10^6^ CFU/μg and ≥5 × 10^9^ CFU/μg of pUC18 respectively). Bacteria were cultured in Lysogeny Broth (LB) medium with appropriate antibiotics (Kanamycin or Ampicillin at 50 or 100 μg ml^−1^ respectively). Qiagen MiniPrep kit was used for DNA isolations from bacteria and Qiagen PCR purification kit and Gel Extraction Kit were used for purifying from PCR reaction or gels (only when using restriction enzymes), following manufacturer instructions. 1% agarose gels were stained with SYBR^®^ Safe and intensities were measured using a BioRad ChemiDoc^TM^ MP Imaging System. 1 Kb DNA ladder (ThermoFisher Scientific) was used for all agarose gel electrophoresis.

### Primers and plasmids

Primers were designed using OligoCalc^54^ and SnapGene® and were obtained from Integrated DNA Technologies (IDT) or Sigma-Aldrich (listed in Supplementary Table S1). All primers were designed to bind template DNA at 60 °C. Plasmids used included pAAV-CW3SL-EGFP (gift from Bong-Kiun Kaang [Addgene plasmid # 61463]), pIRES-mCherry-GluA1, pIRES2-EGFP-GluA2, GluA2 in a CMV-based custom plasmid, pRK5-GluA3, pIRES-EGFP-GluA3, pRK5-GluA4, pGW1-γ2, pIRES2-mCherry, pcDNA4/TO, pIRES-mCherry-GSG1L and pCDNA-pCamKII-EGFP-Homer1c (gift from Andrew Penn).

### PCR reactions and time courses

Unless otherwise stated, 25 μL PCR reactions were performed using Phusion® High-Fidelity DNA polymerase (NEB) with 0.1 μM primers and 1 ng template DNAs (0.5 ng of each for the multi-fragment assembly), according to the following protocol: 30 sec at 95 °C, 18 cycles of 10 sec at 95 °C, 30 sec at 60 °C, 4 min at 72 °C, and a final 5 min extension at 72 °C. Addition of 1 μL FastDigest *Dpn*I enzyme (Thermo Fisher Scientific) was followed by 15 min incubation at 37 °C prior to transformation. For multi-site/multi-fragment procedures, PCR extension time was reduced to 3 minutes, yielding a 1.5 hr PCR reaction.

For insertions, deletions and mutagenesis 2 μL PCR reactions were transformed into 100 μL of RbCl competent cells. For sub-cloning and double modifications 2 μL of *Dpn*I digested sample were transformed in 30 μL of commercial XL10-Gold cells. For multi-site reactions and multi-fragment assemblies 4 μL and 6 μL of PCR mix were transformed into 30 μL and 50 μL of commercial XL10-Gold cells respectively.

All mixtures were incubated for 15 min at 4 °C, followed by a heat shock at 42 °C for 30 sec and recovery at 37 °C for 45 min with 200 μL Super Optimal Broth with Catabolite repression (SOC) medium added. The entire volume was plated onto LB-agar plates with corresponding antibiotics for overnight incubation at 37 °C. Colonies were manually counted (number of colonies reported as Colony-Forming Units per plate (CFU/plate)) and successful plasmid construction was assessed by restriction digestion and/or Sanger sequencing (Beckman Coulter) of colony DNA.

Mutagenesis time courses were performed in multiple 50 μL reactions with 5 ng of DNA template for improved signal at early time points. Whole reactions were removed from the thermocycler every 2 cycles.

### Protocol and primer optimisation

Optimisation of template DNA was performed using 0, 0.1, 0.5, 1, 10 and 50 ng of pRK5-GluA3 template using primers devoid of complementary regions. Length and T_m_ of overlaps was optimised by varying GC content using either increasing length of overlap while keeping temperature constant, or increasing T_m_ while keeping length constant. Experiments were performed three times and average intensities and/or colony numbers are presented together with standard error of the mean.

Enzyme tests included Phusion^®^ High-Fidelity DNA polymerase (New England Biolabs), Pfu Turbo (Agilent), KOD Hot Start Polymerase^®^ (Novagen) and Taq DNA polymerase (Invitrogen). 25 μL PCR reactions were run as previously stated in corresponding manufacturer buffers. All experiments were carried out in triplicate and mean values are reported. PCR product intensities were normalised to the highest intensity product.

## Additional Information

**How to cite this article**: García-Nafría, J. *et al*. IVA cloning: A single-tube universal cloning system exploiting bacterial *In Vivo* Assembly. *Sci. Rep.* **6**, 27459; doi: 10.1038/srep27459 (2016).

## Supplementary Material

Supplementary Information
