# Supplementary material for: IVA cloning: A single-tube universal cloning system exploiting bacterial In Vivo Assembly
Source: Sci Rep. 2016 Jun 6;6:27459. doi: 10.1038/srep27459 (PMC4893743; doi:10.1038/srep27459)
Supplement: Supplementary Information [file srep27459-s1.pdf]

IVA cloning: A single-tube universal cloning system exploiting bacterial *In Vivo* Assembly

Javier García-Nafría[1,*,+], Jake F. Watson[1,+] and Ingo H. Greger[1]

[1] Neurobiology Division, MRC Laboratory of Molecular Biology, Cambridge, CB2 0QH, UK
* jgarcia@mrc-lmb.cam.ac.uk
+ these authors contributed equally to this work

SUPPLEMENTARY MATERIAL

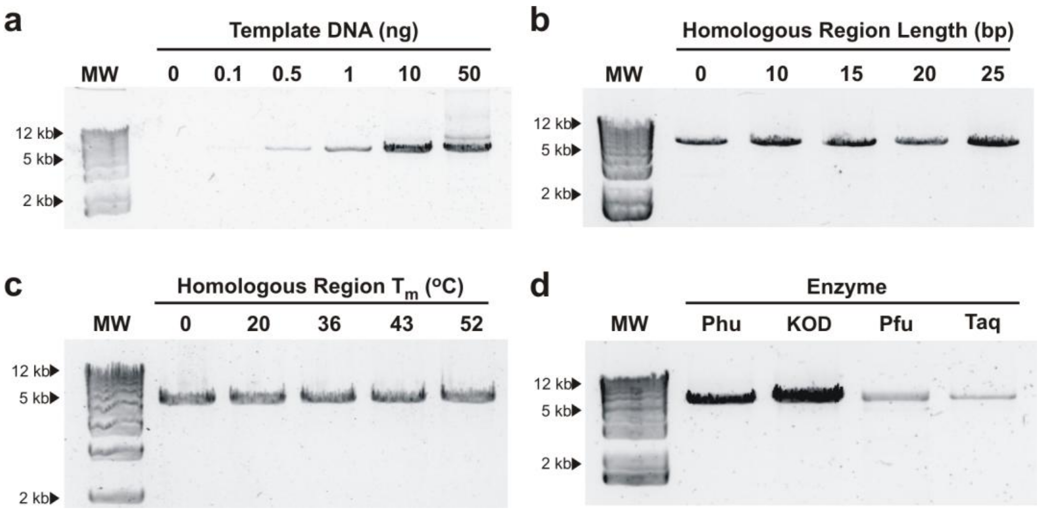

Supplementary Figure S1. Method and primer optimisation. Agarose gels showing PCR amplifications with increasing amount of template DNA **(A)**. Using different homologous region lengths **(B)** and T_m **(C)**, equivalent amplifications are seen in all cases, attributing the differences in colony formation exclusively to recombination efficiency. **(D)** Agarose gel showing difference in amplification efficiency for four polymerases performing the same deletion. (MW = 1 kb Plus Ladder).

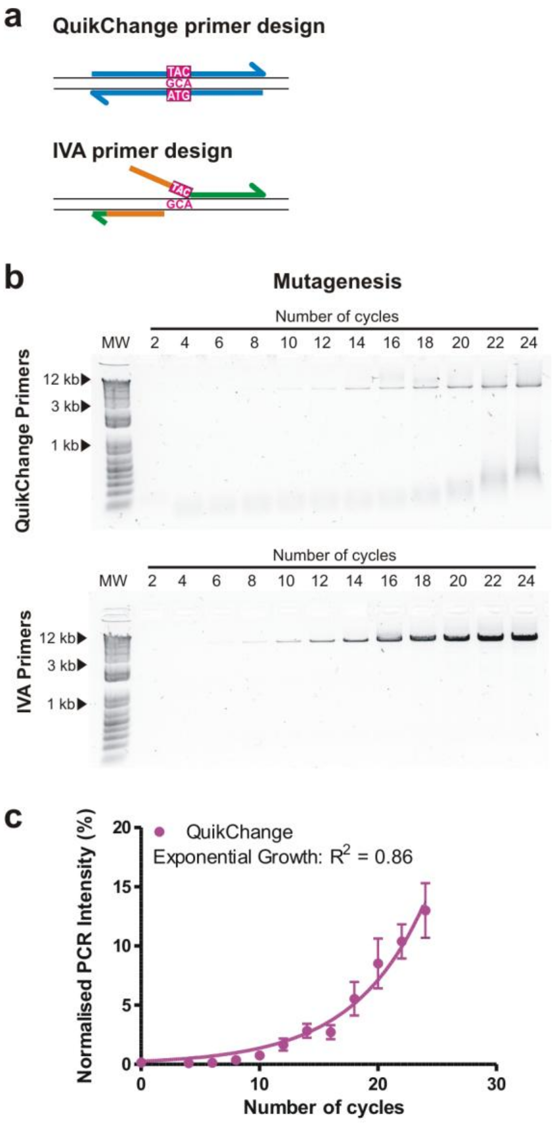

Supplementary Figure S2. Insertion, deletion and mutagenesis amplifications. (A) A comparison of primer design for mutagenesis using QuikChange™ (upper - blue) and IVA (lower - green). **(B)** Comparison of IVA and QuikChange™ amplification rates by examining band intensity every two PCR cycles. Higher intensity and no low molecular weight smearing are seen using IVA primers. (MW = 1 kb Plus Ladder). **(C)** When plotting intensity against cycle number, an exponential curve can be fitted (R squared = 0.86) when using QuikChange™ mutagenesis primers (normalised to IVA cloning).

Supplementary Table S1. List of primers. Homologous regions are shown in italics and mutation codons in bold.

Optimisation

Template opt.		Function
OPT1-F	AATGACAGCTCATCCTCAGAGAACCGG	Amplify pRK5 without recombination
OPT1-R	TCCTCCGTGAGAATGACCCAAAAGCC	

Length opt.		Tm (^{o}C)	Length (bp)	
OPT2-F	*CGCCCGGCGG* AATGACAGCTCATCCTCAGAGAACCGG	40	10	Delete GluA3 NTD region in pRK5
OPT2-R	*CCGCCGGGCG* TCCTCCGTGAGAATGACCCAAAAGCC	40	10	
OPT3-F	*CACGTCCAGAAGAAT* AATGACAGCTCATCCTCAGAGAACCGG	40	15	
OPT3-R	*ATTCTTCTGGACGTG* TCCTCCGTGAGAATGACCCAAAAGCC	40	15	
OPT4-F	*ATTCTTATGGACATTAATTA* AATGACAGCTCATCCTCAGAGAACCGG	40	20	
OPT4-R	*TAATTAATGTCCATAAGAAT* TCCTCCGTGAGAATGACCCAAAAGCC	40	20	
OPT5-F	*ATTATAATATTTACTATATATTATT* AATGACAGCTCATCCTCAGAGAACCGG	40	25	
OPT5-R	*AATAATATATAGTAAATATTATAAT* TCCTCCGTGAGAATGACCCAAAAGCC	40	25	

Tm opt.		GC%	Tm (^{o}C)	
OPT6-F	*ATTATTAATTATTTA* AATGACAGCTCATCCTCAGAGAACCGG	0	20	Delete GluA3 NTD region in pRK5
OPT6-R	*TAAATAATTAATAAT* TCCTCCGTGAGAATGACCCAAAAGCC	0	20	
OPT7-F	*GTCATCAGTTCTTTC* AATGACAGCTCATCCTCAGAGAACCGG	40	36	
OPT7-R	*GAAAGAACTGATGAC* TCCTCCGTGAGAATGACCCAAAAGCC	40	36	
OPT8-F	*GACGTCAGCGTGGTA* AATGACAGCTCATCCTCAGAGAACCGG	60	45	
OPT8-R	*TACCACGCTGACGTC* TCCTCCGTGAGAATGACCCAAAAGCC	60	45	
OPT9-F	*GGCGTCAGCGCGGTC* AATGACAGCTCATCCTCAGAGAACCGG	80	53	
OPT9-R	*GACCGCGCTGACGCC* TCCTCCGTGAGAATGACCCAAAAGCC	80	53	

Delete/Insert		
INS1-F	*GAACAAAAACTCATCTCAGAAGAGGATCTG* TTCCCCAACACCATCAGCATAGGTGG	Inserting myc tag at GluA3 N-terminus in pRK5-GluA3 (IVA)
INS1-R	*TCTTCTGAGATGAGTTTTTGTTC* TCCTCCGTGAGAATGACCCAAAAGCC	
INS2-F	**P**-TCAGAAGAGGATCTG TTCCCCAACACCATCAGCATAGGTGG	Inserting myc tag at GluA3 N-terminus (phosphorylated primers)
INS2-R	**P**-GATGAGTTTTTGTTC TCCTCCGTGAGAATGACCCAAAAGCC	
DEL1-F	*TGGGTCATTCTCACGGAGGA* AATGACAGCTCATCCTCAGAGAACCGG	Deleting GluA3 N-terminal domain in pRK5-GluA3
DEL1-R	TCCTCCGTGAGAATGACCCAAAAGCC	
DEL2-F	*CTGATTTTTGGTGTC* TCTTCTAACAGCATACAGATAGGGGGG	Deleting myc tag at GluA2 N-terminus in pIRES-GluA2
DEL2-R	GACACCAAAAATCAGTCCCCATAAAACAG	
DELINS1-F	*GAAAACCTGTACTTCCAGTCC* ATG GTG AGC AAG GGC GAG GAG CTG	Replacing IRES with linker in pIRES-GluA2 EGFP
DELINS1-R	*GGACTGGAAGTACAGGTTTTC* CTTCATTCGTTTCGCCTCGGCCCTTG	

**Mutagenesis**	**IVA cloning**	
MUT1-F	*CCAGATCGTGAAGCTA* **TGC** AAGAATGGCATCGGGTACCACTACATCC	GluA1 E202C mutation in pIRES-GluA1
MUT1-R	**GCA**TAGCTTCACGATCTGGCCCAGGATG	
MUT2-F	*ACTGAAGCATTCCGT* **TCC**CTTCGGAAGCAGAGGATTGAAATATCCCG	GluA2 N292S mutation in pIRES-GluA2
MUT2-R	GGAACGGAA TGCTTCAGTCATCACTTGGACAG	
MUT3-F	*ACAAATTGTGAGTGTT* **TGC** AAGCATGTCAAAGGCTACCATTATATCATC	GluA4 G208C mutation in pRK5-GluA4
MUT3-R	**GCA**AACACTCACAATTTGTTCTAAAATGTTTTGAAGC	
MUT4-F	*ACCGACTACCTCCAG* **TAG**TCCGCCATCACCCGCATCCCC	γ2 A219STOP mutation in pGW1-γ2
MUT4-R	**CTA**CTGGAGGTAGTCGGTGGCACGG	

	**QuikChange mutagenesis**	
MUT5-F	CCAGATCGTGAAGCTA**TGC**AAGAATGGCATCG	GluA1 E202C mutation in pIRES-GluA1
MUT5-R	CGATGCCATTCTT**GCA**TAGCTTCACGATCTGG	
MUT6-R	GACTGAAGCATTCCGT**TCC**CTTCGGAAGCAGAGG	GluA2 N292S mutation in pIRES-GluA2
MUT6-F	CCTCTGCTTCCGAAG**GGA**ACGGAATGCTTCAGTC	
MUT7-R	GAACAAATTGTGAGTGTT**TGC**AAGCATGTCAAAGGCTAC	GluA4 G208C mutation in pRK5-GluA4
MUT7-F	GTAGCCTTTGACATGCTT**GCA**AACACTCACAATTTGTTC	
MUT8-F	CCGACTACCTCCAG**TAG**TCCGCCATCACCCG	γ2 A219STOP mutation in pGW1-γ2
MUT8-R	CGGGTGATGGCGGA**CTA**CTGGAGGTAGTCGG	

**Subcloning**		
SUB1-F	*ACCGTCAGATCCGCTAGC* ATGAAGACGAGCCGCCGCGGC	Amplifies GSG1L for pIRES vector
SUB1-R	*GATCTGAGTCCGGTAGC* TCACACCCAGTGCCCCAGGACCC	
SUB2-F	GCTACCGGACTCAGATCTCGAGC	Amplifies pIRES vector for GSG1L
SUB2-R	GCTAGCGGATCTGACGGTTCACTAAAC	
SUB3-F	*TGGTACCGAGCTCGGATCC* ATGCAAAAGATTATGCATATTTCTGTCCTCCTTTCTC	Amplifies GluA2 for pcDNA4/TO
SUB3-R	*GTGCTGGATATCTGCAGAATTC* CTAAATTTTAACACTCTCGATGCCATATACGTTGTAAC	
SUB4-F	GAATTCTGCAGATATCCAGCACAGTGGC	Amplifies pcDNA4/TO vector for GluA2
SUB4-R	GGATCCGAGCTCGGTACCAAGCTTAAG	
SUB5-F	*ATCGATAAGCTTGATTCGAGCTAGCC* ACCATGGTGAGCAAGGGCG	Amplifies EGFP-Homer1c for AAV-CW3SL vector
SUB5-R	*CTCGAGATAATCAACCTCTGGATTA* TTAGCTGCATTCTAGTAGCTTGGCCAAATTATCC	

**Multi-site**		
INS3-F	*GACTACAAGGACGACGATGACAAG* TCTTCTAACAGCATACAGATAGGGGGGC	Inserts FLAG at GluA2 N-terminus in pCustom vector
INS3-R	*TCATCGTCGTCCTTGTAGTC* GACACCAAAAATCAGTCCCCATAAAACAGGAGA	
DEL3-F	*TTCCCAGAATTTTGCAACTTAT* AAGGAAGGTTACAACGTATATGGCATCGAGAG	Deletes FLAG at GluA2 C-terminus in pCustom vector
DEL3-R	ATAAGTTGCAAAATTCTGGGAATTCTGCGAGGAAG	

DELINS2-F	*AAGGATGACGACGATAAG* AATGACAGCTCATCCTCAGAGAACCGG	Exchanges GluA3 NTD for FLAG at in pRK5-GluA3
DELINS2-R	ATCGTCGTCATCCTTGTAATCTCCGTGAGA	
SUB3-F	*AGATCGGAAGCGGAAGCGGC* GGGCTGTTTGATCGAGGTGTTCAAATGC	Amplifies γ2 for GluA3-γ2 tandem construct
SUB3-R	*CTTCTGGTGGGAAGGGATCC* TCATACGGGCGTGGTCCGGCGG	
SUB4-F	GGATCCCTTCCCACCAGAAGCATG	Amplifies pRK5-GluA3 vector for GluA3-γ2 tandem construct
SUB4-R	*GCCGCTTCCGCTTCC* GATCTTAACACTTTCTGTTCCATACACGTTGTAG	
**XhoI sites**		
MUT9-F	*ATGGGGCAAAGCGTGCTC* **GAG** GCGGTCTTCTTTTTAGTCCTGGGGC	XhoI site 1 in pRK5-GluA3
MUT9-R	**CTC** GAGCACGCTTTGCCCCATTTTCTTCTG	
MUT10-F	*ACTGGAAAGAGTCATGCATG* **CTC** GAGCCAACATTACAGGTTTCCAGATTGTCAAC	XhoI site 2 in pRK5-GluA3
MUT10-R	**GAG** CATGCATGACTCTTTCCAGTAAAATGTCAGTAAAAC	
MUT11-F	*GCAAGGATGTGATATTTCTC* **GAG** GATCACTTTCTGGGCGCATTGTTGGAG	XhoI site 3 in pRK5-GluA3
MUT11-R	**CTC** GAGAAATATCACATCCTTGCTGCATGAAAGCA	
MUT12-R	*CCTGATGCGGTATTTTCTC* **GAG** ACGCATCTGTGCGGTATTTCACACCG	XhoI site 4 in pRK5-GluA3
MUT12-F	**CTC** GAGAAAATACCGCATCAGGCGCCATTC	
MUT13-R	*ACAAGCTGTGACCGT* **CTC** GAGCTGCATGTGTCAGAGGTTTTCACC	XhoI site 5 in pRK5-GluA3
MUT13-F	**GAG** ACGGTCACAGCTTGTCTGTAAGC	
**Assembly**		
ASS1-F	*GCAGTGAGCGCAACGCAA* TGCTTAGGGTTAGGCGTTTTGCGC	Amplifies CMVtet for Assembly
ASS1-R	*CTATGGAGGTCAAAACAGCG* TCTCTATCACTGATAGGGAGATCTCTATCAC	
ASS2-F	*CGCTGTTTTGACCTCCATAG* ATGGTGAGCAAGGGCGAGGAGC	Amplifies EGFP for Assembly
ASS2-R	*GATGGTGTTGGGGAATCC* CTTGTACAGCTCGTCCATGCCGAG	
ASS3-F	GGATTCCCCAACACCATCAGCATAGG	Amplifies GluA3 for Assembly
ASS3-R	*GCCGCTTCCGCTTCC* GATCTTAACACTTTCTGTTCCATACACGTTGTAG	
ASS4-F	*CGAAGCTTGAGCTCGAG* TCATACGGGCGTGGTCCGGC	Amplifies γ2 for Assembly
ASS4-R	*AGATCGGAAGCGGAAGCGGC* GGGCTGTTTGATCGAGGTGTTCAAATGC	
ASS5-F	TGGCCGCCATGGCCCAACTTG	Amplfies pRK5-GluA4 vector for Assembly
ASS5-R	ATTGCGTTGCGCTCACTGCCCG	
**Library**		
LIB1-F	TGGCCGCCATGGCCCAACTTG	Amplifies pRK5-GluA3 vector for Library
LIB1-F	ATTGCGTTGCGCTCACTGCCCG	
LIB2-F	*GCAGTGAGCGCAACGCAAT* GCTCGCCCGACATTGATTATTGACTAG	Amplifies CMV promoter for Library
LIB2-F	*CTATGGAGGTCAAAACAGCG* AGCTCTGCTTATATAGACCTCCCACCG	

LIB3-F	*GCAGTGAGCGCAACGCAAT* CACTTGTGGACTAAGTTTGTTCGCATCC	Amplifies CamKII promoter for Library
LIB3-F	*CTATGGAGGTCAAAACAGCG* GCTGCCCCCAGAACTAGGGG	
LIB4-F	*CGCTGTTTTGACCTCCATAG* CGAATTCGAATATGCCGTACATCTTTGCC	Amplifies GluA1 for Library from pIRES-GluA1
LIB4-F	*GGGCCATGGCGGCCA* TTACAATCCTGTGGCTCCCAAGGGC	
LIB5-F	*CGCTGTTTTGACCTCCATAG* GCTAGCGGATTCTTCTGCCTTCACTTC	Amplifies GluA2 for Library from pIRES-GluA2
LIB5-F	*GGGCCATGGCGGCCA* CTCGAGGCACTCAGAAGGTTCCTATC	
LIB6-F	*CGCTGTTTTGACCTCCATAG* GAATTCGGCACGAGGTTGCGCC	Amplifies GluA3 for Library from pIRES-GluA3
LIB6-F	*GGGCCATGGCGGCCA* GGATCCCTAGATCTTAACACTTTCTGTTCC	
